# Editorial: Women in neurorobotics

**DOI:** 10.3389/fnbot.2023.1332724

**Published:** 2023-11-28

**Authors:** Mariacarla Staffa, Silvia Tolu, Jiyeon Kang

**Affiliations:** ^1^Department of Science and Technology, University of Naples Parthenope, Naples, Italy; ^2^Department of Electrical and Photonics Engineering, Technical University of Denmark, Lyngby, Denmark; ^3^School of Integrated Technology and AI Graduate School, Gwangju Institute of Science and Technology, Gwangju, Republic of Korea

**Keywords:** neurorobotics, neural mechanism understanding, human robot communication, emotion and cognitive HRI, AI in neurorobotics, women in AI

## 1 Introduction

In today's world, the underrepresentation of women in scientific research is a stark reality. Less than 30% of researchers worldwide are women, a statistic that reveals the persistent gender gap within the scientific community. This gap is exacerbated by long-standing biases and gender stereotypes that discourage girls and women from pursuing careers in science-related fields, with STEM research bearing a significant brunt of these challenges. Recognizing the pivotal role that science plays in shaping our future, it is imperative that we prioritize gender equality in the scientific realm. To change the course of traditional mindsets and foster a more inclusive scientific landscape, we must actively promote gender equality, confront, and dismantle harmful stereotypes, and most importantly, encourage girls and women to pursue STEM careers.

For this reason, we proudly introduced the Research Topic “*Women in neurorobotics*” on the Frontiers in Neurorobotics Journal. Neurorobotics, a multidisciplinary field that melds neuroscience, robotics, and artificial intelligence, offers a glimpse into a future where machines can emulate human-like intelligence and behavior.

This initiative is our commitment to empower, support, and celebrate the contributions of women in Neurorobotics, recognizing that their unique perspectives and talents are essential for advancing the frontiers of scientific knowledge. By showcasing the achievements of women in Neurorobotics, we hope to inspire the next generation of female scientists and foster a more inclusive and diverse community that will drive innovation and progress in this exciting field.

The range of contributions you will find in this collection is nothing short of inspiring. These articles, authored by women who have made significant strides in the field, span the entire spectrum of Neurorobotics research. They cover groundbreaking theory, experimentation, and methodology with applications that tackle some of the most compelling problems of our time.

## 2 Papers in this Research Topic

*Understanding the neural mechanisms of empathy toward robots to shape future applications:* This article, authored by Jenna H. Chin, Kerstin S. Haring, and Pilyoung Kim, provides an overview of modern neuroscience evaluations linking to robot empathy. It evaluates the brain correlates of empathy and caregiving, with a specific emphasis on women. The understanding of these brain correlates can inform the development of social robots with enhanced empathy and caregiving abilities, benefiting various aspects of society, including the transition to parenthood and parenting, where women play a crucial role. The article also discusses some of the barriers women face in the field and underscores the importance of broad representation among researchers (Chin et al.).

*Enactive artificial intelligence: subverting gender norms in human-robot interaction:* This paper, authored by Inês Hipólito, Katie Winkle, and Merete Lie, introduces Enactive Artificial Intelligence (eAI) as a gender-inclusive approach to AI, focusing on the subversion of gender norms within Robot-Human Interaction in AI. The study employs a multidisciplinary framework to explore the intersectionality of gender and technoscience. It reveals the development of four ethical vectors (explainability, fairness, transparency, and auditability) as essential components for promoting gender-inclusive AI. By considering these vectors, AI can align with societal values, promote equity and justice, and create a more just and equitable society for all (Hipólito et al.).

*Continuous joint velocity estimation using CNN-based deep learning for multi-DoF prosthetic wrist for activities of daily living:* by Zixia Meng and Jiyeon Kang, states that myoelectric control of prostheses is a well-established technique, but it often involves isolated movements that do not mirror natural movements during daily activities. This article addresses the need for a control system for multi-degree-of-freedom (DoF) prosthetic arms trained using surface electromyography (sEMG) data collected from activities of daily living (ADL) tasks. It focuses on two major wrist movements, pronation-supination, and dart-throwing movement (DTM), introducing a new wrist control system. The proposed training strategy, “Quick training,” is designed to handle real-world variations such as sensor displacement, muscle fatigue, and sensor contamination. The results, based on data from 24 participants, indicate the effectiveness of this approach, with significant improvements in root mean square error and Pearson correlation values across various ADL tasks (Meng and Kang).

*Classifying human emotions in HRI: applying global optimization model to EEG brain signals:* this article, authored by Mariacarla Staffa, Lorenzo D'Errico, Simone Sansalone, and Maryam Alimardan highlights that significant efforts have been made in the past decade to humanize both the form and function of social robots to increase their acceptance among humans. This study addresses the challenges of emotion recognition using brain-computer interfaces during human-robot interaction. EEG signals were collected from participants interacting with a robot, and machine learning models were trained to classify human emotional responses to the robot's behavior. The results demonstrate the potential to classify emotional responses from EEG signals, opening the door for social robots to comprehend users' emotional states and attribute mental states to them, advancing the field of human-robot interaction (Staffa et al.).

*Social robots as effective language tutors for children: empirical evidence from neuroscience:* This study, authored by Maryam Alimardani, Jesse Duret, Anne-Lise Jouen, and Kazuo Hiraki, explores children's brain responses to robot-assisted language learning. EEG signals were collected from children learning French vocabularies in two groups, one learning from a social robot with narrated French stories and animations, and the other from a display without the robot. The results indicate increased brain synchronization in the theta frequency band in the Robot group, a factor previously associated with success in second language learning. This neuroscientific evidence highlights the effectiveness of social robots as language tutors for children, offering new possibilities for educational technologies (Alimardani et al.).

## 3 Conclusion

The guest editors have been proud to contribute to FRONTIERS platform championing diversity, innovation, and equality. We invite you to explore the impressive research presented in this collection and join us in celebrating the remarkable women who are shaping the future of Neurorobotics.

## Author contributions

MS: Conceptualization, Project administration, Supervision, Writing—original draft, Writing—review & editing. ST: Writing—review & editing. JK: Writing—review & editing.

